# Supplementary material for: Antibiotic prescription patterns in patients with suspected urinary tract infections in Ecuador
Source: PLoS One. 2023 Nov 30;18(11):e0295247. doi: 10.1371/journal.pone.0295247 (PMC10688952; doi:10.1371/journal.pone.0295247)
Supplement: S1 Table — (DOCX) [file pone.0295247.s001.docx]

**S1 Table**

**Variables and Definitions**

| **Variable** | **Definition** | **Scale** | **Indicator** | **Type** |
| --- | --- | --- | --- | --- |
| Level of Health Care | The level or type of healthcare facility where the patient is receiving treatment. | Categorical | Categories (e.g., First, Second) | Nominal |
| Sex of Prescriptor | Gender of the healthcare provider prescribing treatment. | Categorical | Categories (e.g., Male, Female) | Nominal |
| Age of Prescriptor | Age of the healthcare provider prescribing treatment. | Continuous | Years | Quantitative |
| Type of Consultations | Type or nature of medical consultations by type of physician. | Categorical | Categories (e.g., Generalist, Specialist) | Nominal |
| Sex of Patient | Gender of the patient receiving medical treatment. | Categorical | Categories (e.g., Male, Female) | Nominal |
| Age of Patient | Age of the patient receiving medical treatment. | Continuous | Years | Quantitative |
| Education Level of Patient | Educational qualification or level of education of the patient. | Categorical | Categories (e.g., Primary, High School, University) | Nominal |
| Sexual Activity of Patient in the past 12 weeks | Sexual activity of the patient in the past 12 weeks. | Binary | Categories (e.g., Yes, No) | Nominal |
| Comorbidity Presence | Presence or absence of other health conditions or diseases in the patient. | Binary | Presence (Yes/No) | Nominal |
| Type of Comorbidity | Specific type or nature of the comorbidity if present. | Categorical | Categories (e.g., Diabetes, Hypertension, None) | Nominal |
| Temperature | Body temperature of the patient during the examination. | Continuous | Degrees Celsius | Quantitative |
| Fever Presence | Presence or absence of fever in the patient. | Binary | Presence (Yes/No) | Nominal |
| Dysuria | Pain or discomfort experienced during urination. | Binary | Presence (Yes/No) | Nominal |
| Frequency of Urination | Frequency of urination reported by the patient. | Binary | Presence (Yes/No) | Nominal |
| Urgency of Urination | Urgency or immediate need to urinate reported by the patient. | Binary | Presence (Yes/No) | Nominal |
| Lumbar Pain | Pain in the lower back region reported by the patient. | Binary | Presence (Yes/No) | Nominal |
| Hematuria | Presence of blood in the urine. | Binary | Presence (Yes/No) | Nominal |
| Vaginal Discharge | Abnormal vaginal discharge reported by female patients. | Binary | Presence (Yes/No) | Nominal |
| Urethral Discharge | Abnormal urethral discharge reported by male patients. | Binary | Presence (Yes/No) | Nominal |
| ICD-10 Code | International Classification of Diseases (ICD-10) code for the diagnosed condition. | Categorical | Alphanumeric code ( N10: Acute pyelonephritis,  N300: Acute cystitis,  N390: Urinary tract infection, site not specified) | Nominal |
| Clinical Syndrome UTI | Clinical syndrome indicating Urinary Tract Infection (UTI). | Categorical | Categories (e.g., Cystitis in female patients, Pyelonephritis in female patients  UTI_male, Cystitis in male patients, Pyelonephritis in male patients) | Nominal |
| Urine Dipstick Result | Presence of urine dipstick test. | Binary | Categories (e.g., Yes/ No) | Nominal |
| Nitrite Presence | Presence or absence of nitrites in the urine sample. | Binary | Presence (Positive/Negative) | Nominal |
| Leucocytes Presence | Presence or absence of leucocytes in the urine sample. | Binary | Presence (Positive/Negative) | Nominal |
| Bacteria Presence | Presence or absence of bacteria in the urine sample. | Binary | Presence (Positive/Negative) | Nominal |
| GRAM Stain Result | Result of the GRAM stain test conducted on the urine sample. | Categorical | Categories (e.g., Positive, Negative) | Nominal |
| Urine Culture | Result of the urine culture test. | Categorical | Categories (e.g., Positive, Negative) | Nominal |
| Antibiotic Prescription | Prescription of antibiotics for the patient. | Binary | Presence (Yes/No) | Nominal |
| Type of Antibiotic | Specific type or name of the prescribed antibiotic. | Categorical | Categories (e.g., Penicillin, Amoxicillin, etc.) | Nominal |
| Duration of Antibiotic Treatment in Days | Number of days the patient is prescribed antibiotics. | Continuous | Number of days | Quantitative |
| Appropriate Antibiotic Criteria for antibiotic prescription | Compliance with criteria used to determine the appropriateness of antibiotic prescription according to study protocol. | Binary | Met criteria (Yes/No) | Nominal |

Dataset is available in: Sánchez, Xavier (2023), “Antibiotic Prescription Patterns in Patients with Suspected Urinary Tract Infection in Ecuador”, Mendeley Data, V2, doi: 10.17632/styystf2nj.2
