# Supplementary material for: Antibiotic prescription patterns in patients with suspected urinary tract infections in Ecuador
Source: PLoS One. 2023 Nov 30;18(11):e0295247. doi: 10.1371/journal.pone.0295247 (PMC10688952; doi:10.1371/journal.pone.0295247)
Supplement: Attachment — Submitted filename: 23-05-07-Jimbo 2023 subm Pone-Review-23-05-07-Jimbo 2023 subm-PONE-D-23-12797-Antibiotic prescription patterns in patients with susp UTI in Ecuador.docx [file pone.0295247.s002.docx]

Jimbo 2023 subm-Review-PONE-D-23-12797-Antibiotic prescription patterns in patients with suspected UTI in Ecuador
