# Supplementary material for: Antibiotic prescription patterns in patients with suspected urinary tract infections in Ecuador
Source: PLoS One. 2023 Nov 30;18(11):e0295247. doi: 10.1371/journal.pone.0295247 (PMC10688952; doi:10.1371/journal.pone.0295247)
Supplement: Attachment — Submitted filename: Response to Reviewers.docx [file pone.0295247.s003.docx]

***Point by point response letter.***

*Additional Journal Requirements:*

***1. Please ensure that your manuscript meets PLOS ONE's style requirements, including those for file naming. The PLOS ONE style templates can be found at***

***https://journals.plos.org/plosone/s/file?id=wjVg/PLOSOne_formatting_sample_main_body.pdf and***

***https://journals.plos.org/plosone/s/file?id=ba62/PLOSOne_formatting_sample_title_authors_affiliations.pdf***

Thank you for your guidance regarding the style requirements for manuscript submission to PLOS ONE. We have carefully reviewed and followed the provided style templates for both the main body and the title, authors, and affiliations sections of our manuscript. Our submission now adheres to PLOS ONE's formatting guidelines. Your assistance is greatly appreciated.

***2. In the ethics statement in the Methods, you have specified that participant consent was obtained. Please provide additional details regarding how this consent was documented and witnessed, and state what participants consented to***

We appreciate your keen attention to the ethics statement in our Methods section. We've taken your suggestion to heart and have now expanded upon the details regarding how participant consent was documented and witnessed, as well as providing a comprehensive description of the elements to which participants provided their consent. The revised manuscript now contains this enhanced information to ensure transparency and thoroughness in our reporting. Thank you for your valuable input in refining our work.

***3. We noticed you have some minor occurrence of overlapping text with the following previous publication(s), which needs to be addressed:***

***https://www.tandfonline.com/doi/abs/10.1080/00325481.2017.1246055?journalCode=ipgm20***

***https://www.ncbi.nlm.nih.gov/books/NBK557569/***

***https://www.sciencedirect.com/science/article/abs/pii/S0009898117302292?via%3Dihub***

***In your revision ensure you cite all your sources (including your own works), and quote or rephrase any duplicated text outside the methods section. Further consideration is dependent on these concerns being addressed.***

Thank you for bringing this to our attention. We sincerely apologize for the oversight and any inconvenience caused. We are committed to addressing the issue of overlapping text and ensuring proper citation of all relevant sources. We will carefully review the manuscript to identify any duplicated text outside the methods section and either quote or rephrase it accordingly. Your feedback is greatly appreciated, and we will make the necessary revisions to ensure the integrity and originality of our manuscript.

***4. We note that you have stated that you will provide repository information for your data at acceptance. Should your manuscript be accepted for publication, we will hold it until you provide the relevant accession numbers or DOIs necessary to access your data. If you wish to make changes to your Data Availability statement, please describe these changes in your cover letter and we will update your Data Availability statement to reflect the information you provide.***

Thank you for your message. We appreciate your focus on data availability. We have indeed taken the required measures to furnish repository information for our data. The pertinent access details for our data have been submitted, and we are dedicated to ensuring the provision of the necessary accession numbers. Cite: Sánchez, Xavier (2023), “Antibiotic Prescription Patterns in Patients with Suspected Urinary Tract Infection in Ecuador”, Mendeley Data, V1, doi: 10.17632/styystf2nj.1 (https://data.mendeley.com/preview/styystf2nj).

***5. Your ethics statement should only appear in the Methods section of your manuscript. If your ethics statement is written in any section besides the Methods, please delete it from any other section.***

Thank you for pointing out the location of the ethics statement. We've reviewed and corrected the manuscript to ensure that the ethics statement appears exclusively within the Methods section as per your guidance. We appreciate your attention to detail and have taken steps to align with the proper formatting.

**Reviewer #1:**

***“Now you could discuss more specifically especially for the first two groups, what is appropriate and what is not appropriate. Unfortunately N390 is a black box exept general recommendations and considerations about local resistance, because it is not known, how many complicated UTI incl pyelonephritis cases are included in each antibiotic prescription. In summary: the reader has problems to comprehend, which antibiotic under which consideration you consider appropriate or inappropriate. Of course, nitrofurantoin and oral fosfomycin would be the preferred antibiotics for uncomplicated cystitis. Fluoroquionolones should not more prescribed at all for UTI unless for specific complicated UTI, e.g. prostatitis. Still, we do not know, what cases are included in N390.”***

We greatly appreciate the valuable feedback provided by the reviewer. In response to these insightful points, we have implemented several improvements in the revised manuscript.

Firstly, we have included explicit definitions of what we consider to be appropriate prescription, aiming to enhance clarity for readers. Lines: 169-172

Secondly, we have enhanced the description of the methodology we employed to identify UTI cases, providing a more comprehensive understanding of our approach. Lines: 139-148

We acknowledge the limitation introduced by the CIE code N390 and its impact on our study's ability to disaggregate data. This limitation arises from the retrospective nature of our methodology. We have, however, taken steps to clarify the criteria we utilized and have duly addressed this limitation in the discussion section. Lines: 405-409.

We sincerely hope that the reviewer recognizes the importance of this limitation in the context of our study design. Once again, we express our gratitude for the reviewer's suggestions, which have undeniably contributed to the improvement of our manuscript.

**Reviewer #2:**

***“The authors present an interesting and up-to-date cross-sectional look at UTI prescription patterns in Ecuador. The overall results certainly seem to be consistent with my own observations from clinical practice, and add the interesting observation that prescriber age, lack of use of POC testing, and care by a generalist may be associated with increased risk of an inappropriate prescription - all information that may be useful for antimicrobial stewardship programs.***

***I do think a few points need to be addressed before proceeding with publication:***

***1) The extremely high prescription rate (99%) and specific wording of inclusion criteria (p6, line 127) leave me wondering - was treatment expressly part of inclusion criteria? (I don't think this is a problem per se - just worth clarifying as the question of appropriate antibiotic treatment is quite different than appropriate evaluation of urinary symptoms).”***

We are grateful for the reviewer's valuable input. In our study, we initiated the evaluation process based on diagnoses provided by medical practitioners. Specifically, we employed UTI codes to identify cases and subsequently assessed the appropriateness of antibiotic treatment in relation to the diagnosed UTI and the selected antibiotic. Notably, the inclusion criteria did not encompass any form of treatment. Moreover, we rigorously assessed the suitability of immediate antibiotic prescriptions using clinical parameters extracted from the medical records, thereby evaluating whether the specified criteria were met or not. To enhance clarity and comprehension, we have taken measures to enhance the delineation of our inclusion and exclusion criteria within the manuscript. Lines: 139-148. Your feedback has been instrumental in refining the presentation of our study's methodology.

***“2) Regarding the included list of ICD10 codes (p 6 line 130-131): These codes certainly capture the more certain diagnoses. However, prior research has suggested variable performance in diagnosis-specific vs symptomatic/syndromic ICD10 codes for identifying patients evaluated for UTI (https://www.ncbi.nlm.nih.gov/pmc/articles/PMC7558992/). Would suggest the authors provide some citation or justification for the specific list chosen. If current list is kept, may need to add to limitations some statement that this ICD10 code list does risk some selection bias in targeting patients. For example, if patients with "dysuria" were also included, it is possible the rate of inappropriate treatment might have been even higher.”***

We sincerely appreciate the reviewer's insightful observation and valuable suggestion regarding the selection of ICD10 codes for identifying patients evaluated for UTI. We acknowledge the importance of considering both diagnosis-specific and symptomatic/syndromic codes in such studies. Unfortunately, it's worth noting that in Ecuador, we do not utilize the ICD-10-CM (International Classification of Diseases, Tenth Revision, Clinical Modification) for our electronic health records. Consequently, the identification of UTI cases based on symptom-related codes is not a feasible option in our context. Nonetheless, we have provided a more comprehensive explanation of the inclusion and exclusion criteria in the methodology section. Lines: 139-148.

While we recognize that the ICD-10 codes without symptom codification may carry limitations, we would like to emphasize that, given the retrospective nature of our study, this approach was the most viable means of identifying UTI cases within our setting. We fully concur with the reviewer's understanding that these codes, without symptom-based classifications, may be prone to errors of omission and misclassification.

We have taken your suggestion to heart and have included a discussion of this limitation in our manuscript. Additionally, we have referenced the study you provided, which evaluated this concern, in our discussion section. We believe that this reference will enrich the dialogue on the potential implications of our chosen methodology. Lines 401-412

Once again, we extend our gratitude to the reviewer for their valuable feedback, which has undoubtedly contributed to enhancing the transparency and rigor of our study's methodology and discussion.

***“3) On sample size calculations (p 6 lines 138-141): Though the formula was nicely provided, I am still uncertain how exactly this was used to choose sample size. Please clarify if possible - which outcome was this powered on, and what was the targeted power or precision?”***

We appreciate the valuable insights from the reviewer. In response, we have enhanced the clarity and comprehensiveness of the sample calculation explanation within the methodology section. Lines: 162-164.

***“4) On cohort selection (p 6 line 144): Related to the sample size calculations, how was the cohort actually selected? Was it a random sample to meet goal sample size? Or consecutive cases? All cases?”***

Expanding on the previous recommendation, we have continued to enhance the clarity and comprehensiveness of the explanation and procedure for both sample calculation and sample selection within the methodology section. Lines: 152-161.

We value the insights provided by the reviewer, and the revisions implemented have significantly improved our article.

***“5) On assessment of antibiotic appropriateness: From a stewardship perspective, appropriateness may refer to whether indication, antibiotic selection, or duration were reasonable. In this case, it looks like appropriateness is adjudicated solely on whether an indication was appropriate. If so, please stage explicitly.”***

Thank you for your insightful comment. We have made adjustments to clarify that our assessment of antibiotic appropriateness primarily focuses on the indication for use. We appreciate your stewardship perspective and have addressed this concern in our revised manuscript. Lines: 169-172.

***“6) On stepwise selection method (p 7, line 164): A few more details would be helpful to understand model selection:***

***- What were the candidate variables considered and how were these selected?***

***- I note comorbidity is only included as a yes/no variable- was any consideration given to inclusion of just comorbidities associated with urinary symptoms?***

***- What rules were used in the stepwise selection and what parameter was used to assess "best performing" model?”***

Thank you for your invaluable insights into our methodology. Your suggestions to enhance the clarity of our model selection process are greatly appreciated. We have diligently revised the manuscript to incorporate additional details addressing your queries.

Regarding the selection of candidate variables: We have now provided a comprehensive clarification regarding the meticulous process through which we selected candidate variables. These variables were carefully chosen based on their theoretical significance and were further refined using preliminary bivariate logistic regression models. Lines: 188-190.

Your keen observation about comorbidity inclusion is astute. While comorbidity was captured as a binary variable (yes/no), we acknowledge the significance of considering comorbidities specifically associated with urinary symptoms. However, certain conditions potentially linked to a higher risk of urinary tract infections, such as cystocele, urolithiasis, benign prostatic hyperplasia, paraplegia, and diabetes, were encountered infrequently. Consequently, due to their limited occurrence, we chose to incorporate them as a binary variable to ensure their consideration without risking statistical validity. We hope you understand and appreciate this necessary compromise, which we have transparently addressed in our revised manuscript.

In terms of the stepwise selection process and identifying the "best performing" model: We have provided a more detailed description of our step-by-step selection approach, and we have explained that we use the Akaike Information Criterion (AIC) as the determining parameter. This criterion adeptly strikes a balance between model fit and complexity, leading us to a well-optimized model choice. Lines: 194-197.

We extend our heartfelt gratitude for your thorough review, which has undoubtedly elevated the quality and transparency of our research. Your invaluable input is immensely appreciated and contributes to the robustness of our study.

***“7) "First level of health care" (p 9, line 173, but also other places throughout text): Not all readers may be familiar with this term. Please define at first use.”***

Thank you for your feedback. We have incorporated the definitions of "first level of healthcare" and "second level of healthcare" in the design and setting section of the manuscript to enhance clarity for all readers. Lines: 125-129.

***“8) Text density on p 9: In general, many of the descriptive statistics on p 9 could best be left in the tables so as not to be redundant or text heavy.”***

Thank you for your valuable feedback. We appreciate your suggestion regarding text density on page 9. In the revised manuscript, we have made improvements by optimizing the presentation of descriptive statistics, ensuring a balanced and non-redundant text. This enhances the clarity and readability of our study.

***“9) Clarity on p 10, line 200-201:***

***- Does this mean >1 bacterium per HPF was considered a positive result for microscopic bacteria? If so, this seems overly sensitivity and prone to over-calling UTI.***

***- "Gram negative stain bacteria in all cases" - I am uncertain about the intended meaning of this line. Please reword if possible.”***

Thank you for your valuable feedback. We apologize for any confusion caused. We have revised the wording to clarify that the description pertains to the components of the microscopy test conducted in the cases, rather than establishing diagnostic criteria. Additionally, we have corrected the statement to accurately reflect that Gram stains were performed in all tested cases. Lines: 227-228. We appreciate your attention to these points and have made the necessary adjustments in the revised manuscript.

***“10) On variable selection: I notice prescriber age was converted to a binary variable splitting at age 40. Was there an a priori reason for this selection?***

***- If there is not a good reason for this split point, was prescriber age assessed as either a continuous variable or ordinal groups like patient age was?”***

In our analysis, we observed that the prescriber's age variable exhibited a mean age of 37.62 years, with a standard deviation of 8.95, encompassing an age range from 25 to 60 years. The distribution of age values displayed a notable symmetry. In light of these observations, we made a deliberate a priori decision to transform this continuous variable into a binary form, with the split occurring at age 40. This choice was rooted in both statistical considerations and practical advantages. From a statistical standpoint, while retaining age as a continuous variable remains viable, the dichotomization approach offers improved result interpretability and promotes a more focused inquiry. Moreover, it effectively addresses potential non-linearities that may arise if age were employed as a continuous predictor. Additionally, the selection of age 40 was guided by our consideration of sample size and statistical power. By dividing the age variable at this threshold, we attain a balanced distribution of prescribers across distinct age groups, thereby heightening our capacity to discern meaningful associations. This strategic choice also furnishes a robust basis for conducting subgroup analyses and exploring potential age-related effect modifications.

In summary, the conversion of the prescriber's age variable into a binary format at age 40 was underpinned by meticulous theoretical reasoning and grounded in empirical insights from the dataset. By adopting this methodological approach, we have fortified the robustness of our study's design, amplifying our potential to unearth valuable insights into the intricate interplay between prescriber age and prescription patterns. We sincerely appreciate your understanding of the rationale behind our decision.

***“11) Assessment for collinearity: It comes to mind that certain providers - by nature of their specialty - might be associated with particular patient types. Did the authors assess their regression models for the possibility of collinearity among included variables?”***

We appreciate your consideration of potential collinearity due to providers' specialties and patient types. While we employed established methods to assess collinearity, it's noteworthy that the levels of care in our study, spanning both generalists and specialists, demonstrate a relatively balanced distribution across specialties. This context helps mitigate significant collinearity concerns.

Our approach involved meticulous variable selection. The strategy employed is outlined in the Statistical Analysis section, where we confirm the absence of collinearity among variables. Ultimately, while the nature of specialties was a factor in our analysis, the even distribution of care levels attenuates the impact of collinearity on our regression models.

We value your meticulous observations and have incorporated the relevant explanations in the revised manuscript. Lines: 190-191.

***“12) Caution with epidemiologic wording on p 13, line 254: Would be careful about stating "Our data show that almost 90% of cases develop in women" as this study was a cross sectional one aimed at assessing UTI prescribing appropriateness - and is not actually an epidemiologic survey. E.g., it did not actually assess relative incidence in men/women.***

***- Would reword to say something akin to: "Nearly 90% of patients seeking evaluation for UTI were women..." etc”***

Thank you for your valuable feedback. We appreciate your thoughtful consideration of the epidemiologic wording concern. We have carefully revised the sentence in question to align with your suggestion. Lines: 282-284. Your input has contributed to enhancing the clarity and accuracy of our manuscript.

***“13) Use of terms "firstline" or "secondline": A bit of a technicality, but the guideline cited (Gupta et al 2011) uses the term "recommended" rather than firstline. Consider rewording for consistency.”***

Thank you for your thoughtful observation. We appreciate your attention to detail. We have taken your feedback into account and have made the necessary revisions to ensure consistency in our terminology. The term "recommended" has been appropriately used throughout the manuscript to align with the guideline cited (Gupta et al., 2011). We value your input and have worked to enhance the clarity and accuracy of our manuscript.

***“14) Regarding emphasis of conclusions:***

***- Suggest rewording abstract to refer to appropriate or inappropriate (rather than "adequate") prescription rate”***

Thank you for your valuable suggestion. We have thoroughly reviewed your feedback and implemented the required revisions in the abstract and across the manuscript. The terms "adequate" and "inadequate" have been substituted with "appropriate" and "inappropriate" to precisely represent the prescription rate. We are grateful for your meticulous input, which has contributed to enhancing the clarity of our manuscript.

***“15) Causality assessed in p 17, line 347-348: Can the authors provide specific evidence for their statement that uncertainty from insufficient diagnostic capacity influenced treatment in their study? I think this is an important point - and could be strengthened if they could list for example what proportion of their prescribers had access to UA, culture, etc?”***

Thank you for your valuable feedback. We acknowledge the importance of providing specific evidence to support our statement regarding the influence of diagnostic capacity on treatment decisions. Unfortunately, due to limitations in sharing detailed characteristics of the individual health centers, which may involve sensitive information, we are unable to provide specific details about center characteristics.

However, in response to your suggestion, we have enhanced our discussion of the broader characteristics of the healthcare system in Ecuador to better support our assertion. Our revised manuscript now provides a more comprehensive overview of the country's healthcare system, highlighting the challenges related to the lack of resources and the accessibility of laboratory tests for patients. Lines: 380-388.

We hope you understand the complexities of sharing certain center-specific details while still striving to strengthen our argument with the available information. We appreciate your thoughtful consideration of this aspect of our study and have taken measures to address it.
