# Supplementary material for: Antibiotic prescription patterns in patients with suspected urinary tract infections in Ecuador
Source: PLoS One. 2023 Nov 30;18(11):e0295247. doi: 10.1371/journal.pone.0295247 (PMC10688952; doi:10.1371/journal.pone.0295247)
Supplement: Attachment — Submitted filename: Response to Reviewers.docx [file pone.0295247.s004.docx]

***Point by point response letter.***

**Reviewer #1:**

***“Here is my 1. Question: The reader would like to see a table according to the categories mentioned in Chapter „Assessment of appropriateness of antibiotic prescription“. Because most of the patients had UTI not specified, the authors could state, that they tried to specify the UTIs according to symptoms, comorbidities, gender, and positive urine dipstick tests.***

***Then probably the five categories mentioned in this chapter could correspond to***

***1. uncomplicated cystitis in women***

***2. uncomplicated pyelonephritis in women***

***3. complicated UTI (obviously in women, because the next category says. „any male“)***

***4. complicated UTI in male patients***

***5. Here the reader would like to know, what the authors assumed in case no urine dipstick test was performed or the test was negative besides symptoms were present.***

***After the authors have clarified the consequence of category 5 (are the patients without or negative dipstick test included or excluded from further analysis or maybe still categorized according to the categories 1-4), the the reader would like to see a similar table as Table 2 with the following UTI categories (according to symptoms and comorbidities):***

***1. uncomplicated cystitis in women***

***2. uncomplicated pyelonephritis in women***

***3. complicated UTI in women***

***4. UTI in male patients***

***5. Total***

***Such a table would be more informative than table 2 according to ICD-10, where 81.85% of patients had UTI, not specified. Therefore, such a table should be added.”***

We greatly appreciate the valuable suggestions provided by the reviewer. The recommendation to classify cases according to symptoms rather than relying solely on ICD-10 diagnosis codes is indeed insightful. The ICD-10 code N390, as rightly pointed out by the reviewer, can be somewhat of a "black box" that requires further clarification as it does not distinguish between cystitis and pyelonephritis cases. In response to this, we have taken the following actions to enhance the clarity and accuracy of our study:

1) Methodological Clarification: In our revised manuscript, we have incorporated an explanation within the Methods section detailing that the same criteria used to assess appropriateness were also employed to classify cases according to clinical diagnosis. This approach helps to bridge the gap between clinical symptoms and ICD-10 codes, providing a more comprehensive understanding of the patient population. Lines 181-183.

2) New Classification Categories: To address the issue of classification, we have introduced four distinct categories in our analysis, differentiating between cystitis and pyelonephritis cases in both women and men. While we recognize the relevance of sub-classifications, such as distinguishing between complicated and uncomplicated cases, regrettably, we did not have access to all the necessary information to perform such a detailed classification. Nevertheless, it is noteworthy that, based solely on the comorbidities identified in the records, the number of cases that could be classified as complicated appears to be quite limited. We hope this clarification helps shed light on our approach to addressing this limitation. Lines: 221-223.

The introduction of the new Table 2, as suggested by the reviewer, has significantly improved the informativeness of our manuscript. Moreover, it has revealed important insights, including instances of antibiotic misuse, such as the use of nitrofurantoin in pyelonephritis cases. See new table 2: Table 2. Antibiotic use by type of clinical diagnosis

In summary, we want to express our gratitude to the reviewer for their constructive feedback. We believe that the changes we have implemented have substantially enhanced the quality and clarity of our study. We hope that these modifications address the concerns raised and contribute positively to the overall impact and relevance of our research.

***“This problematic (most patients had UTI, not specified9 should be discussed also in limitations.”***

We greatly appreciate the valuable suggestion. We have now incorporated a discussion of the limitations associated with the ICD-10 code N390 in our research. Lines: 404-412.

***“Urine cultures were performed only 10.64%, most in cases with pyelonephritis (ok), but 37.85% also in UTI, not specified. Here the additional table with clinical specification could also be helpful.”***

Thank you for your valuable suggestions. While we haven't added a new table, we have provided clarification on the results in lines 234–238.

***“Only 62.5% of urine cultures were positive according to the definition used (>100.000 CFU/ml). Here the authors could also discuss, e.g. in chapter limitations, that it is not known whether the patients were pretreated with antibiotics – or that lower CFU/ml can also be significant, especially in uncomplicated cystitis in women [1,2]”***

Thank you for your valuable suggestion. We have incorporated these aspects into the discussion section, specifically in lines 329-337. Additionally, the references you suggested have been included in the manuscript. We appreciate your input, as it has significantly enhanced the quality of our manuscript.
