# Supplementary material for: Antibiotic prescription patterns in patients with suspected urinary tract infections in Ecuador
Source: PLoS One. 2023 Nov 30;18(11):e0295247. doi: 10.1371/journal.pone.0295247 (PMC10688952; doi:10.1371/journal.pone.0295247)
Supplement: Attachment — Submitted filename: Response to Reviewers.docx [file pone.0295247.s005.docx]

***Point by point response letter.***

***Reviewer #2:***

***“No addition comments from prior - I believe the authors have addressed all reviewers' points to the fullest extent possible”***

We appreciate the feedback provided by the reviewer and are pleased to acknowledge that he believes the authors have adequately addressed all points raised. His input has been invaluable in enhancing the quality of our manuscript.

***Reviewer #3:***

***“The manuscript in current form should be acceptable in current shape. The manuscript in current shape is seemed to be properly formatted and incorporated with comments. There is no need for further changes, therefore I recommned this manuscript to be accepted by the editor.”***

We express our sincere gratitude for the positive feedback of the reviewer on the current state of our manuscript. His thorough evaluation and endorsement for its acceptance by the editor are truly appreciated.

***Reviewer #4:***

***“1) Data availability issue: the data are made available by the authors but, in the way they are shared, they are not helpful to reproduce the main analysis. First, a code-book description and explanation of each variable (please rename all variables in English) with the specification of the coding used is missing and it seems that some variables are not provided. Please also specify if they refer to prescribers or patients.”***

Thank you for your feedback and careful evaluation of our data availability. We sincerely appreciate your time and attention to detail in assessing our manuscript.

In response to your concern, we have provided a comprehensive data set in English, including the names and meanings of the variables, along with the corresponding codes for each variable used in the analysis. We have ensured that the data set Version 2 is structured to enable a clear understanding of each variable and its relevance to the study. We have also specified whether the variables refer to prescribers or patients, enhancing the clarity of our data set.

Please note that we have incorporated only the variables that are directly relevant to the study, ensuring that the provided data set aligns precisely with the scope of our research. The data is available in: <https://data.mendeley.com/datasets/styystf2nj/2>

We hope these modifications address your concerns adequately. Thank you once again for your valuable input; your feedback has significantly contributed to the enhancement of our work.

***“2) In the methods, it should be clarified that the sample does not contain repeated measurements within patients. The main outcome and variables collected should be better specified and defined. In fact, it is not clear if the main outcome is the prescription pattern or the appropriateness of the prescription (I understand the second). This should be better highlighted and each variable listed specifying how it is defined (categorical, continuous ...). Otherwise, a more detailed data description should be provided as Supplemental. Please also refer the reader to the data shared (in a better understandable way).”***

Thank you for your invaluable suggestions. We have duly clarified in the methods section (lines 167-168) that the sample excludes repeated measurements within patients. Regarding the main outcomes, we appreciate your insight, and in the methods section, specifically under the subheading "Design and Setting", in lines 124-126, indicates that our study aimed to analyze both antibiotic prescription patterns and the appropriateness of prescriptions concerning the clinical features of the patients.

Understanding the need for detailed variable descriptions, we have taken your suggestion seriously. We have included a comprehensive breakdown of each variable, specifying their definitions and categorizations, in Supplementary Material 1 (referenced in lines 190-191). Your meticulous review has significantly contributed to refining our manuscript, and we sincerely appreciate your valuable feedback.

***“3) The statistical analysis does not take into account the correlation of patients followed by the same prescriber. In fact, it's not sufficient to adjust for prescribers' characteristics but also to adjust for the prescribers as random effects. That is, instead of a simple multivariable logistic regression, a mixed regression model should be performed. Moreover, the mean(sd) of the number of patients seen by the same prescriber is not reported. In the discussion, lines 303-304, page 15, it's reported that general practitioners and specialists were responsible for almost the same number of diagnoses of UTI but this is not evidenced in the results. Moreover, the authors wrote "bivariate analysis" but this is in fact "univariate regression analysis". Please correct it in line 192, methods and line 272, results”***

Mixed-effects regression models are commonly used in longitudinal studies to analyze repeated data from the same subjects over time. These models are particularly suitable for handling the correlation between repeated observations of the same individual. Our study had a cross-sectional design, where observations were made at a single point in time, and subjects were not followed over time. Consequently, our sample does not contain repeated data for each individual. Since mixed-effects regression models are specifically designed for repeated data, their use in cross-sectional studies is neither common nor appropriate, as the data structure does not align with the nature of these models. Taking these considerations into account, we have decided to maintain the analysis through multivariable logistic regression. We hope the reviewer understands the rationale behind this decision.

Reporting mean and standard deviation of the number of patients seen by each prescriber is not pertinent to the outcomes we analyze. Our analysis aligns with methodologies employed in studies conducted across various contexts. Despite conducting a thorough search for additional studies to comprehend the possible significance of this result, we did not find any research that considered this specific aspect.

We have rephrased the sentence that may have been confusing, indicating that general practitioners and specialists were responsible for almost the same number of UTI diagnoses in lines 308-309.

We apologize for the confusion regarding the terminology used. We have corrected the terms and changed "bivariate analysis" to "univariate regression analysis" in lines 195 and 277. We appreciate the reviewer’s attention to detail regarding our study.

***“4) The authors have not appropriately addressed the previous reviewers' comment. If a table with the subgroup specification uncomplicated/complicated was not possible due to the lack of information, a subgroup analysis, based on the element/factors which defined appropriateness of prescriptions should be given. In fact, it's not clear how many ciprofloxacin prescriptions, for example, were appropriate or not.”***

We appreciate the reviewer's attention to our study. Regarding the categorization of urinary tract infections as complicated or uncomplicated, we faced challenges due to numerous clinical factors (such as the lack of information about anatomical abnormalities of the urinary tract) and microbiological factors (like the presence of multi-resistant bacteria) that are essential for such classification. Given the complexity of these variables and the nature of our study design, precise categorization proved unfeasible. These limitations were duly acknowledged by previous reviewers and were understood, these constraints have been detailed in lines 427-436. Furthermore, performing a subgroup analysis would have similar limitations since this information is not available to ensure accurate classification, compromising the accuracy of our data.

Our methodology was specifically designed to assess the appropriateness of antibiotic prescriptions based on clinical features, as detailed in the "Assessment of appropriateness of antibiotic prescription" section of our methods. Unlike evaluating specific antibiotic choices, our focus was on the clinical conditions justifying a prescription, aligning with established clinical guidelines. These guidelines recommend first, and second-line antibiotics based on clinical features; hence, the decision to align with recommendations.

For example, the reviewer's mention of evaluating the appropriateness of ciprofloxacin (or any type of antibiotic) prescriptions underscores our point. In current medical practice, ciprofloxacin is no longer the recommended first-line treatment for cystitis due to evolving antibiotic resistance patterns. Consequently, any prescriptions of this antibiotic would be considered inappropriate. However, the choice of antibiotics relies on localized antibiotic resistance data, an aspect recommended in guidelines, a crucial aspect that we extensively address in our discussion, we contrast guidelines recommendations with our local patterns of antibiotic resistance. Finally, the factors influencing practitioners' antibiotic selection were not assessed and fall outside the scope of our research.

We hope this clarifies our approach. Once again, we express our gratitude for the reviewer's thorough analysis, which has enhanced the depth of our study.

***5) Results presentation is not systematic and clear. The authors should present more results in Tables and not in plain text, so that the reader could better follow and understand where reported results come from. In Table 1, please also report the count (%) of the patients with no comorbidities. The reader should catch immediately all relevant information. Please rephrase line 225 on page 11: it is not clear to which % they refer or maybe the authors forgot to report the number % of the second level of care. In Table 1 please correct "Age (SD)" moving (SD) after mean.***

We sincerely appreciate the meticulous feedback provided by the reviewer. It's important to note that some of these observations had been previously raised and successfully resolved based on previous suggestions from other esteemed reviewers. We value this collective scrutiny, which significantly contributes to the enhancement of our manuscript.

In terms of the presentation of results, we aimed to strike a balance between tables and text, aiming for a comprehensive and reader-friendly approach. While certain data complexities necessitated textual explanations, we have diligently incorporated as much information as possible within tables to ensure clarity.

Regarding the concern about the percentage of UTIs in the second level, we have included this information in lines 209-201, precisely detailing its context for readers' understanding.

Moreover, we have thoroughly reviewed Table 1, implementing the necessary adjustments. The count and percentage of patients without comorbidities have been included to facilitate immediate comprehension. Additionally, we have restructured the presentation of age, placing (SD) after the mean, in accordance with the reviewer's recommendation.

We trust that these refinements have further polished the systematic and coherent presentation of our results, thanks to your invaluable guidance.

***6) The discussion should be better rewritten. First, a summary of the main results: please first state the more important ones (related to the outcome) and then the secondary ones. Then, discuss each point (the reader would appreciate if subheading were used). The novelty of this study is not highlighted: the reader does not catch what this study adds to the existing literature. No mention is given about the % range of prescription of each antibiotic (nitrofurantoin, fluoroquinolones...) in the existing literature and in the international context. A range should be provided, since the prescription pattern is a main outcome and the reader should understand how much the % of prescriptions of fluoroquinolones (appropriate/not appropriate) found in the study, for example, differs compared to the range found in the literature. Moreover, not being able to differentiate between complicated/uncomplicated should be stated among the limitations because this limits the interpretation of the results compared to the literature, since the authors referred to studies of appropriateness for uncomplicated UTI.***

We appreciate the reviewer's input. We have made modifications to the discussion section's structure, aiming to balance previous feedback from reviewers who endorsed the existing structure with the need to contextualize the results concerning the main outcomes: antibiotic prescription patterns and appropriateness. We hope the reviewer understands our reasoning; our intent was not to dismiss prior suggestions from other respected reviewers. Additionally, we have incorporated subheadings for better understanding, as the reviewer suggest keeping the guidelines submission of the journal.

In lines 354-362, we have incorporated the predominant antibiotic usage ranges in UTIs as suggested, providing readers with a reference point for our results. It's crucial to emphasize once more that our study evaluates the appropriateness of antibiotic prescriptions based on clinical features. We compare the use of first and second-line antibiotics for UTIs in alignment with guidelines and local antibiotic resistance patterns, taking the specific local context into account. This aspect represents the novelty of our approach—striving to contextualize clinical features within the specific setting where physicians practice. Furthermore, our study stands as one of the few reporting these outcomes in South America.

In addressing the study's limitations related to the challenge of accurately distinguishing between complicated and uncomplicated UTIs, we elaborated on these aspects in lines 427-436."

We greatly value the constructive feedback provided by the reviewer, and we assure that all comments have been thoroughly considered to enhance the quality of our manuscript.

***7) Minor: line 199 please correct "utilized" with "use". That's sounds better.***

Thanks for the suggestion. We have changed the word utilized for used. Line: 203
