# Supplementary material for: Antibiotic prescription patterns in patients with suspected urinary tract infections in Ecuador
Source: PLoS One. 2023 Nov 30;18(11):e0295247. doi: 10.1371/journal.pone.0295247 (PMC10688952; doi:10.1371/journal.pone.0295247)
Supplement: Attachment — Submitted filename: Response to Reviewers.docx [file pone.0295247.s006.docx]

***Point by point response letter.***

***Reviewer #4:***

***“The authors did not address one of my previous comments regarding the use of mixed models. The authors should perform a model with prescribers as random effects because in the study there are repeated measurements within prescribers, though it is true that in the study there are no repeated measurements within patients and the study is no longitudinal. However it is also true that in the study prescribers characteristics are included in the model (this is correct) but patients followed by the same prescriber are correlated and this should be accounted for in the adjusted model. Maybe the results would not change so much and the conclusions will remain the same. Anyway the authors should perform this analysis. The rest of the manuscript sounds good and once performed this analysis and correct the methods and results accordingly, the manuscript would be suitable for publication (unless other issues would not come out after this analysis).”***

Thank you for your insightful comments and recommendations. We acknowledge the importance of accounting for the correlation among patients followed by the same prescriber. In response to your suggestion, we have employed a mixed effects logistic regression model, incorporating a random effect for health professionals (prescribers), as detailed in the Methods section (lines 191-209). This adjustment has been meticulously applied in our updated analysis, which is now presented in Table 4.

Upon careful examination of the outcomes, we noted minor variations in some decimal estimations, with no substantial changes in the overall results. Crucially, our conclusions have remained consistent despite these adjustments. We have also updated the abstract to reflect these new findings, ensuring the manuscript's accuracy and completeness.

We genuinely appreciate your thoughtful critique, which has undoubtedly enhanced the robustness of our analysis and the final version of our manuscript.
